# Normal Island: COVID-19, Border Control, and Viral Nationalism in UK Public Health Discourse

**DOI:** 10.1177/13607804211049464

**Published:** 2021-11-25

**Authors:** Des Fitzgerald

**Affiliations:** University of Exeter, UK

**Keywords:** borders, COVID-19, independent Sage, nationalism, public health, scientific advice

## Abstract

In this contribution, I present emergent analysis of a preoccupation with managing COVID-19 through border control, among non-Governmental public health actors and commentators. Through a reading of statements, tweets, and interviews from the ‘Independent Sage’ group – individually and collectively – I show how the language of border control, and of maintaining immunity within the national boundaries of the UK, has been a notable theme in the group’s analysis. To theorize this emphasis, I draw comparison with the phenomenon of ‘green nationalism’, in which the urgency of climate action has been turned to overtly nationalistic ends; I sketch the outlines of what I call ‘viral nationalism,’ a political ecology that understands the pandemic as an event occurring differentially between nation states, and thus sees pandemic management as, inter alia, a work of involuntary detention at securitized borders. I conclude with some general remarks on the relationship between public health, immunity, and national feeling in the UK.

On 14 June 2021, Anthony Costello, Director of the UCL Institute for Global Health, previously Director of Maternal, Child, and Adolescent Health at the WHO, appeared on Sky News (2021) – where he lamented the success of UK’s COVID-19 ‘test and trace’ system. ‘You know’, Costello said, ‘we’re in a war. We should have an army on the ground, and we should protect our airspace from invading infections’ (Sky News, 2021).

The use of war metaphors in situations of infectious disease is not new, and has long been critiqued in medical sociology (Larson et al., 2005). In this article, however, I want to take Professor’s Costello’s statement seriously, and at face value, as a claim about what kind of event the pandemic actually is and how we should handle it. I propose in what follows that Costello’s central image – public health as defence against external threat – is, in fact, prevalent in much epidemiological discourse around the COVID-19 pandemic, particularly among the Independent Sage group, of which Costello is a co-founder. I argue that this imagery suggests a striking attachment to securitized national borders including the explicit use of those borders to a protect or defend some national space, among many public health commentators. I use the term ‘viral’ nationalism to theorise this attachment, and I conclude by indicating some of this phenomenon’s central features.

The UK government’s Scientific Advisory Group for Emergencies (Sage) was activated for COVID-19 in January 2020. In the early days of the pandemic, amid much public discussion about whether the conservative government was following an adequately scientific strategy, there was significant disquiet about Sage’s lack of transparency (neither membership nor minutes of meetings were public), perhaps a hangover of the low-key conspiratorialism that had come to characterize much centre-left politics in the wake of the Brexit referendum (Grayling, 2018; Sample and Mason, 2020). Indeed, it was even rumoured in the newspapers that the prime minister’s Chief Advisor, Dominic Cummings, truly a phantasmatic figure for much of liberal England, was an attendee at Sage meetings (Tyler, 2020). In this context, initially 12 scientists, chaired by Sir David King (a former government Chief Scientific Advisor) constituted themselves as ‘Independent Sage’ – an unofficial group that would act as a public shadow of the official body (Baker, 2020). Independent Sage was founded around three broad convictions: (1) that the government’s strategy was likely inadequate to contain the pandemic, and not well rooted in the scientific evidence (Independent Sage, 2020a); (2) that this failure was partly because the government’s scientific advice was tainted by political calculation (Davis, 2020); (3) that transparency of analysis and advice were central to resolving this problem (Baker, 2020).

Through regular self-issued reports, a weekly ‘briefing’ on YouTube (the Independent Sage channel has 118,500 subscribers at the time of writing), a strong collective and individual social media presence, as well as regular mainstream media appearances, Independent Sage has been highly successful at carving out an alternative, semi-formal space for public discussion and analysis of COVID-19 data and strategy. Individual members also regularly discuss the pandemic on Twitter, generally receiving significant attention. Indeed, one might say that ‘indie sage’, as the group also terms itself, came into being with a keen eye for such attention: from the beginning, the group seemed to project a sharply critical and pessimistic general outlook on the pandemic, a stance well met by a downbeat and somewhat paranoid public mood vis-à-vis the government’s capacity and intentions.

At the heart of Independent Sage’s critique of UK government strategy, as well as its more broadly pessimistic outlook, is the idea that this strategy has been fatally compromised by political, rather than purely scientific, calculation (see Cassidy, 2020; Smallman, 2020). Despite this conviction that science and politics must be strictly separated, however, Independent Sage itself has been regularly identified in the press, often by Conservative-supporting outlets, as a group on the political left (Baker, 2020). Indeed, the day after Independent Sage formed, they were dismissed by the *Daily Mail* as a ‘left wing cabal’ and ‘very biased’ (Adams, 2020). *The Sun* later decried the ‘left-wing bias of scientists on Independent Sage’ while the television presenter, Richard Madeley, quizzed one member, Susan Michie, on whether her analysis was influenced by her membership of the Communist Party of Britain (Griffiths, 2021; Lewis, 2021). This accusation of partiality is perhaps not helped by the occasional presence of Jonathan Ashworth, Labour Shadow Secretary of State for Health – whose criticisms of government policy have sometimes resonate with Independent Sage’s analysis – at the group’s briefings.

In any event, the desire to cut science from politics has clearly proven more complicated than resonated Independent Sage had anticipated. I say this not to be snide, but rather to contextualize one of the most distinctive elements of the group’s analysis, which many would associate not with the left, but rather with much more reactionary political movements, and this is the group’s firm emphasis on border control. Indeed, at the first Independent Sage meeting, border control was emphasized by Gabriel Scally, a physician, former regional director of public health in south west England, and visiting professor at Bristol University. Scally said ‘There’s one item I wanted to raise, please . . . And that is about border controls’. He continues,

we should be looking at our ports, our airports, and our eurotunnel stops, train stops, from the point of view of what port health regulations and what port health choices we’ve got in terms of *protecting us from importing cases* as we get down to a small number of cases ourselves. (Independent Sage, 2020d, emphasis added)

Notably, the issue was little taken up in the rest of the meeting. However, a subsequent report in the *Guardian* emphasized the meeting’s interest in ‘the potential benefits to harnessing the island status of the UK and Ireland, as countries such as New Zealand have done, and developing new port health policies’ (Davis, 2020). This is a strange claim, given that the UK and Ireland, unlike New Zealand, comprise two different states. Nonetheless one can see an image taking shape: wealthy, defensible European or former British Dominion islands, who eliminate COVID-19 within their own borders, and are then protected from the rest of the planet by ‘port health regulations’.

Through 2020 and 2021, border control and international travel seemed to become more prominent in Independent Sage’s pronouncements. For example, the issue was absent from the group’s ‘emergency six-week plan’ in October 2020 as cases rose in the UK (Independent Sage, 2020b); it was equally absent in a four-point ‘emergency statement’ issued in December 2020, as it became increasingly clear that new restrictions were imminent (Independent Sage, 2020c). A month later, however, the group issued a report on the ‘geographic spread of the virus’ (the only ‘geography’ considered was travel into and around the UK; Independent Sage, 2021a). This report called for ‘managed isolation of travellers’ (managed meant no longer voluntary) with travellers themselves to meet the costs, except for ‘UK citizens [note: not UK residents] who were able to show that their travel was non-optional and that they lacked the means to afford managed isolation’. Strikingly, the report went on to compare poor border control in a pandemic to ‘mopping up water from the floor of your house without fixing the hole in the roof that let water enter in the first place’. This comparison (between isolating human beings ‘under official supervision’ at the border and mopping up unwanted water) was repeated by Gabriel Scally in the *Daily Telegraph* the same day, and Independent Sage’s official Twitter account then tweeted the quote (Newey and Rigby, 2021). In February 2021, by which time the UK’s vaccine rollout was well underway, a call for ‘strict control of borders and limits on international travel’ appeared as the final point in a five-point plan for ‘sustainable suppression’ of the virus (Independent Sage, 2021b). By May 2021, with the UK vaccine programme a marked success, Independent Sage issued a statement on dealing with the new Delta variant, where border control had risen to priority number two (Independent Sage, 2021c). The number one recommendation was global vaccination, with national-level measures only appearing after these two. In other words, in mid-2021 COVID-19 was becoming understood as largely a problem of the world beyond Britain’s borders.

In public statements from members of Independent Sage, border control is also a frequent topic. On Twitter, Christina Pagel (2020, 2021a, 2021b, 2021c), while arguing for ‘strict border controls’ has referred to the UK having a ‘rubbish border policy’, a ‘leaky border policy’, and a ‘shitty border policy’. Kit Yates (2021) argued in the *Huffington Post* that ‘our borders’ are ‘one of [sic.] the most important weapons we have in the fight against Covid’. Gabriel Scally (2021a, 2021b) wrote on Twitter: ‘the only thing that brings this virus to our shores is human bodies . . . The only vehicle for carriage and transmission of the virus is the human body. It’s not medicines, it’s not foodstuffs, it’s not animals – it’s humans’. Zubaida Haque (2017, 2021), one-time interim director of the Runnymede Trust who had previously tweeted criticism of medical personnel being compelled to carry out immigration checks, called for ‘comprehensive and watertight international restrictions’. And Susan Michie (2021) argued that a travel ‘red list’ (i.e. a list of particular countries from which travellers are subject to stricter border measures) was not enough and that instead ‘universal border controls’ were necessary.

Related questions of nationality and citizenship also surfaced in discussions of global vaccine supplies: in a long twitter thread about the UK’s ongoing ‘public health failure’ in July 2021, for example, Anthony Costello (2021) lamented that amid a global vaccine shortage, the UK, unlike ‘every other wealthy country’ that was vaccinating its children, intended to give ‘kids vaccine to Africa [sic.]’ (It is perhaps worth noting here that Costello’s title is ‘Professor of International Child Health’^1^). Others who are not members of Independent Sage, but who are similarly critical of the UK government response, and who have expressed support for the group’s work, have also discussed borders. In January 2021, for example, Devi Sridhar, a member of the Scottish government’s COVID-19 advisory group told the *New Statesman*: ‘It makes no sense to me how you have a government that ran on taking back control of our borders and at a time when you actually need it, from a public health framework to protect the people living in Britain, you don’t use it’ (Eaton, 2021). Strikingly, the same image was used three weeks later by Jonathan Ashworth, who told LBC (2021) it was

ironic that Boris Johnson and [Home Secretary] Priti Patel apparently one of their big motivations is to take back control of our borders, but when we need them to take back control of our borders they didn’t do it to keep us safe.

Here, the overlapping political and geological fantasies of Brexit, seem to be incorporated, wholescale, into the response to COVID-19.

How and why did a group of public health experts and related scholars, broadly identified with the left, become preoccupied with border control to this extent? How did it become obvious within a certain strand of critical public health discourse that that COVID-19 was a problem that the UK should manage through a system of involuntary detention at securitized international borders? Members of Independent Sage themselves would perhaps argue that they advocate only for temporary measures to bring the pandemic under control – thus, indeed, to actually shorten the overall period of restriction. But the idea that one can make such claims for a specific and limited purpose, and then simply return to some more liberal system, all while staying safely removed from the deeply unpleasant, and indeed racist, politics of the border that has predominated in Britain for some decades, is surely quite naive. It is noteworthy that New Zealand, for example, often the paradigmatic case of this strategy, recently announced that it would never return to pre-pandemic migration polices, and would focus, in particular, on permanently excluding ‘low skilled’ migrants (McClure, 2021). The sociologist, Alexandre White (2018), has recently argued for understanding such developments through what he calls ‘epidemic orientalism’ – a historical discursive formation that has long situated ‘the colonial world and the colonized as a permanent, perpetual vector of disease, and the colonizer and his world as the permanently, potential victims of that threat’ (p. 50).^2^ For White, understanding the apprehension of infectious disease in Euro-American countries means understanding how those countries have come to constitute their ideas of themselves in relation to colonial and post-colonial worlds. One cannot simply make innocent epidemiological claims as if these histories do not exist – nor can one presume that these histories are merely historical. As Angela Mitropoulos (2020) put it at the very beginning of this pandemic: there remains political value in quarantine – a value that is ‘grounded in and fosters the stigmatization of groups of people through a territorial-national, and therefore racialized, association with a disease’.

My goal in this article is thus not to engage in a debate about the imagined epidemiological merits of border control. Instead I want to think about how a moment of biological and political crisis may effloresce into a distinctive form of border-fever – in Britain and elsewhere. To expand my account, I want to sketch a comparison to the climate movement, where imaginaries around COVID-19 have also drawn on statements and ideas that, pre-pandemic, would have been associated with more explicitly reactionary political movements.

On 24 March 2020, the Twitter account of the East Midlands branch of Extinction Rebellion posted images of a sticker on a lamp-post. The sticker read: ‘corona is the cure, humans are the virus’ (Cross, 2021). Amid an outcry, the national organization of Extinction Rebellion distanced themselves from the message, with representatives assuring media that the account was likely fake. This claim seemed uncertain: *ITV News* reported that the main Extinction Rebellion Twitter account had followed the East Midlands account until the appearance of the message (Cross, 2021). But one might ask why in any event people with apparently far-right views would purport to represent Extinction Rebellion, Indeed, the fact that at least some fascists see the Extinction Rebellion logo as a useful vehicle for their views (see also Lock, 2020) has so far prompted less introspection among climate activists than perhaps it might.

There is a context here: since its emergence in 2018, Extinction Rebellion has been criticized not only for the overwhelming whiteness of its support, but for mobilizing that whiteness, politically, in its strategy of mass arrest (Gandhi, 2019). The group has also been criticized for raising the spectre of mass migration as a negative consequence of ecological catastrophe, while courting the good opinion of the police (Shand-Baptiste, 2019). Indeed the group once called on the police to ‘leave peaceful protesters alone and focus on knife crime instead’ (Gayle, 2019). Rupert Read (2014), once a spokesperson for the group, and a philosopher at the University of East Anglia, argued some years ago in *The Ecologist* that it was critical to ‘rein in immigration’ and ‘re-localis[e] our economies and societies’.^3^ In 2019, the German branch of Extinction Rebellion distanced itself from Roger Hallam, one of the group’s founders, after he referred to the holocaust as ‘just another fuckery in human history’ (Baynes, 2019).

Let me expand this scene a little: at the same time as the ‘corona is the cure’ poster appeared in March 2020, another series of viral images appeared online, this time depicting the apparent reappearance of various animals in different European cities, as COVID-19 restrictions kept humans from the streets. ‘Here’s an unexpected side effect of the pandemic’, wrote one Twitter user, ‘the water’s [sic.] flowing through the canals of Venice is clear for the first time in forever. The fish are visible, the swans returned’. Another went: ‘Boars in the middle of my hometown, dolphins in the port of Cagliari . . . Nature is reclaiming its spaces during quarantine in Italy’. A third said, ‘Nature just hit the reset button on us’ (Wray, 2020; see Daly, 2020 for a debunking).

Of course there is a lightness to this animal fantasia. For the avoidance of doubt, there is nothing bad or sinister about being charmed by animals in cities! Nonetheless, what these stories also capture, I suggest, is a latent form of post-human desire that has animated a surprisingly large amount of public feeling through the pandemic, that is, a desire for human space, and in particular for urban space, to be at least partly cleansed of pathological humans. The virality of such stories seemed to indicate a strange, millenarian, and even, I would say, sometimes rather violent collective pleasure in seeing towns and cities reclaimed by nature’s innocent creatures. Again, I am not saying that anyone posting such stories thinks this, but it is important to recall here that there is a long history of environmental and conservation movements espousing everything from broadly anti-migrant sentiment to a general suspicion of urban modernity and the humans who constitute it (Bramwell, 1989; Taylor, 2016). At its darker edges, much of this feeling can be affiliated to what Andreas Malm and the Zetkin Collective (2021) have lately called ‘green nationalism’ – a ‘belief in the protection of the white nation as protection of nature . . . [in which] ecological crisis is not denied but enlisted as a reason to fortify borders and keep aliens out; this would imply a break with [climate] denial and a rebranding of various nationalist policies as the remedy’.

My claim here is that if we wish to make sense of the – in my reading – reactionary rhetoric of borders and bodies that animates so much public health rhetoric around COVID-19, and if we also want to make sense of the political difficulty in actually identifying such ideas as, indeed, reactionary, then we may find it useful to diffract that rhetoric through an account of how at least some parts of the environmental movement have, for some years now, become entangled in ideas and images associated with what might minimally be described as overtly nationalist movements. How, in the wake of such an account, would we sketch the outlines of some future “viral nationalism” that might effloresce, or attain wider legitimacy, as the current pandemic continues, or a new one takes hold? How would we begin to account for a novel political affect in which a collective sense of the pandemic as both an environmental catastrophe and a drama of the border is held together? Here, as a starting-point for discussion, I sketch four preliminary features: (1) viral nationalism would take the individual nation’s legal and political boundary as the primary site of infection control; it would pay intense attention to the border as a site of contamination, and evince an easy willingness to promote policed, involuntary containment at that border; (2) viral nationalism would emphasize an approach to infection control that makes international movement largely impossible for many people, especially people in the Global South, for indefinite periods of time; viral nationalism would thus also be defined by a general indifference to, or a gestural interest in, specific problems faced by, for example, migrants and refugees; (3) in its public pronouncements, which are central to its self-constitution, viral nationalism would have at least occasional recourse to forms of rhetoric, metaphor, and imagery that are violent, militaristic, or dehumanizing, and would be unapologetic in the face of critique of such imagery; (4) as a phenomenon of European and Anglophone worlds, viral nationalism would be always deeply embedded in colonial histories, and operate as an exercise of power between wealthy, extractive countries, and, for example, former countries of empire.

To be clear, I am not at all arguing that anyone associated with Independent Sage, or anyone quoted here, subscribes to such claims. Indeed, I am certain that they do not. What I offer here, rather, is an interpretive apparatus that formalizes a wider public feeling about the relationship between the pandemic and the border - an apparatus that seems to me important for a critical analysis of public health discussion, in the media and elsewhere, as this discussion has come to conjure the COVID-19 pandemic as a particular kind of problem, with a particular kind of solution.

The phrase ‘normal island’ has lately become a staple of UK social media discourse; it marks a weary dissent from the sometimes comically jingoist and authoritarian tendencies that animate so much public debate in contemporary Britain. At the risk of being po-faced, however, I suggest that this phrase, which puts normativity and insularity next to one, is worth taking seriously. The precise sense of a troubled insular psycho-ecology – what Paul Gilroy (2005) once called the ‘fantastic structure of the melancholic island race’ – has long underwritten a great deal of national feeling in the UK (p. 102). And if the arrival of public health to this scene is not new, still the affective structure of British nationalism has received striking new impetus during the pandemic. Thus, I suggest that understanding an emphasis on the border within a nominally left epidemiological discourse on pandemic control may help us to conjure the wider political ecology of a country in the throes of Brexit, COVID-19, and, soon, a Scottish independence debate – a moment in which the speculative geography and biology of a cut-off, immunized island race will take on renewed potency. I am arguing here for an emergent public feeling, grounded in a collective environmental and epidemiological anxiety in which Britain, as a naturalized biological community, an island of immunity barricaded against an unliveable planet, might yet the ground of a new and distinctive syndrome, which I call viral nationalism.

## Notes

1. In Costello’s defence, he went on to argue for solving the global vaccination shortage by simply sharing the patent – still, I find the rhetoric used in the course of his argument striking.

2. I am here quoting from White’s PhD thesis, though his ‘epidemic orientalism’ is also the topic of a book manuscript in preparation.

3. It should be noted that that article now appears to have been deleted; Read insists that readings of it that see him as in favour of migration control are wrong, rather that he is simply ‘unconvinced by the case for the complete abolition of border controls’ (Read, 2019).
